# Paramagnetic Effects in NMR Spectroscopy of Transition-Metal Complexes: Principles and Chemical Concepts

**DOI:** 10.1021/acs.accounts.3c00786

**Published:** 2024-04-30

**Authors:** Jan Novotny, Stanislav Komorovsky, Radek Marek

**Affiliations:** † CEITEC – Central European Institute of Technology, Masaryk University, Kamenice 5, CZ-625 00 Brno, Czechia; ‡ Department of Chemistry, Faculty of Science, Masaryk University, Kamenice 5, CZ-625 00 Brno, Czechia; § Institute of Inorganic Chemistry, Slovak Academy of Sciences, Dúbravská cesta 9, SK-84536 Bratislava, Slovakia

## Abstract

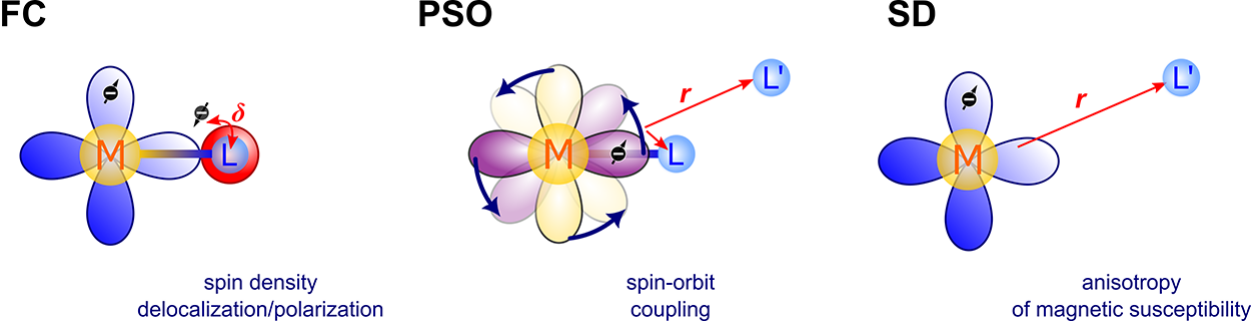

Magnetic resonance techniques represent a fundamental class of spectroscopic methods used in physics, chemistry, biology, and medicine. Electron paramagnetic resonance (EPR) is an extremely powerful technique for characterizing systems with an open-shell electronic nature, whereas nuclear magnetic resonance (NMR) has traditionally been used to investigate diamagnetic (closed-shell) systems. However, these two techniques are tightly connected by the electron–nucleus hyperfine interaction operating in paramagnetic (open-shell) systems. Hyperfine interaction of the nuclear spin with unpaired electron(s) induces large temperature-dependent shifts of nuclear resonance frequencies that are designated as hyperfine NMR shifts (δ^HF^).

Three fundamental physical mechanisms shape the total hyperfine interaction: Fermi-contact, paramagnetic spin–orbit, and spin–dipolar. The corresponding hyperfine NMR contributions can be interpreted in terms of through-bond and through-space effects. In this Account, we provide an elemental theory behind the hyperfine interaction and NMR shifts and describe recent progress in understanding the structural and electronic principles underlying individual hyperfine terms.

The Fermi-contact (FC) mechanism reflects the propagation of electron-spin density throughout the molecule and is proportional to the spin density at the nuclear position. As the imbalance in spin density can be thought of as originating at the paramagnetic metal center and being propagated to the observed nucleus via chemical bonds, FC is an excellent indicator of the bond character. The paramagnetic spin–orbit (PSO) mechanism originates in the orbital current density generated by the spin–orbit coupling interaction at the metal center. The PSO mechanism of the ligand NMR shift then reflects the transmission of the spin polarization through bonds, similar to the FC mechanism, but it also makes a substantial through-space contribution in long-range situations. In contrast, the spin–dipolar (SD) mechanism is relatively unimportant at short-range with significant spin polarization on the spectator atom. The PSO and SD mechanisms combine at long-range to form the so-called pseudocontact shift, traditionally used as a structural and dynamics probe in paramagnetic NMR (pNMR). Note that the PSO and SD terms both contribute to the isotropic NMR shift only at the relativistic spin–orbit level of theory.

We demonstrate the advantages of calculating and analyzing the NMR shifts at relativistic two- and four-component levels of theory and present analytical tools and approaches based on perturbation theory. We show that paramagnetic NMR effects can be interpreted by spin-delocalization and spin-polarization mechanisms related to chemical bond concepts of electron conjugation in π-space and hyperconjugation in σ-space in the framework of the molecular orbital (MO) theory. Further, we discuss the effects of environment (supramolecular interactions, solvent, and crystal packing) and demonstrate applications of hyperfine shifts in determining the structure of paramagnetic Ru(III) compounds and their supramolecular host–guest complexes with macrocycles.

In conclusion, we provide a short overview of possible pNMR applications in the analysis of spectra and electronic structure and perspectives in this field for a general chemical audience.

## Key References

- NovotnýJ.; SojkaM.; KomorovskyS.; NečasM.; MarekR.Interpreting the paramagnetic NMR spectra of potential Ru(III) metallodrugs: Synergy between experiment and relativistic DFT calculations. J. Am. Chem. Soc.2016, 138, 8432–8445.27312929 10.1021/jacs.6b02749(1) Through-bond (Fermi-contact) ligand hyperfine NMR contributions related to propagation of spin density from paramagnetic metal center to ligands are very sensitive to solvent effects and enable experimental NMR data to be interpreted.
- NovotnýJ.; JeremiasL.; NimaxP. R.; KomorovskyS.; HeinmaaI.; MarekR.Crystal and Substituent Effects on Paramagnetic NMR Shifts in Transition-Metal Complexes. Inorg. Chem.2021, 60, 9368–9377.34133172 10.1021/acs.inorgchem.1c00204PMC9597657(2) Through-bond hyperfine interactions are related to the electron configurations of central atoms in transition-metal complexes, and the signs and magnitudes of hyperfine shifts are interpreted in terms of electron π-conjugation and σ-hyperconjugation delocalization.
- BoraP. L.; NovotnýJ.; RuudK.; KomorovskyS.; MarekR.Electron-Spin Structure and Metal–Ligand Bonding in Open-Shell Systems from Relativistic EPR and NMR: A Case Study of Square-Planar Iridium Catalysts. J. Chem. Theory Comput.2019, 15, 201–214.30485092 10.1021/acs.jctc.8b00914(3) Signs of electronic g-tensor and paramagnetic spin–orbit contribution to hyperfine NMR shifts are interpreted by the molecular-orbital theory and linked to the electron configuration at the transition-metal atom.
- NovotnýJ.; ChybaJ.; HruzíkováA.; PikulováP.; KursitA.; KnorM.; MarkováK.; MarekJ.; JurčekP.; JurčekO.; MarekR.Flipping hosts in hyperfine fields of paramagnetic guests. Cell Rep. Phys. Sci.2023, 4, 101461.(4) Through-space hyperfine shifts in electronic doublet systems, given by the paramagnetic spin–orbit and spin–dipolar contributions, are used to determine relative orientation in supramolecular host–guest assemblies.

## 1 Introduction

Nuclear magnetic resonance (NMR) spectroscopy has been utilized for decades to solve various problems in the chemistry of transition-metal (TM) compounds. Myriad papers have described its application to diamagnetic (closed-shell) systems, and this topic has been extensively reviewed.^5,6^ The NMR dependencies for ligand atoms in diamagnetic TM compounds resemble those known for organic molecules except for those situations where relativistic heavy-atom effects on the NMR resonances of neighboring light atoms play a significant role.^7^

The situation is fundamentally different for paramagnetic (open-shell) systems, where the NMR resonances are greatly shifted due to the imbalanced magnetic contributions of α/β electron spin at finite temperature and broadened by paramagnetic relaxation.^8−10^ Paramagnetic NMR (pNMR) shifts are excellent indicators of the underlying (particularly long-range) electron–nucleus hyperfine interactions governed by the molecular structure. Within the approximations discussed in Section 2, the total pNMR shift of atom L can be expressed as the sum of the orbital and hyperfine (also known as Curie) contributions in eq 1:^11,12^
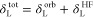
1The orbital contribution to the NMR shift (δ_L_^orb^, approximately temperature-independent) is related to the NMR shift of its diamagnetic counterpart. In contrast, the hyperfine contribution (δ_L_^HF^, temperature-dependent) originates from the presence of unpaired electrons in the molecule and results in NMR resonances in large spectral regions. Three physical mechanisms contribute to δ_L_^HF^: Fermi-contact, spin–dipolar, and paramagnetic spin–orbit. We provide an elemental theory behind the hyperfine shifts and describe recent progress in understanding the structural and electronic principles underlying the individual hyperfine mechanisms.

## 2 Theory

The quantum mechanical theory of pNMR has been known for more than 50 years, but only recently has it been realized that the proper starting point for any temperature-dependent molecular property (including pNMR) is the Helmholtz free energy and its derivatives.^12,13^ Paramagnetic NMR theory was originally developed within an exact-state formalism, however it is possible to express the pNMR theory in terms of parameters of the EPR effective spin Hamiltonian.^14,15^ This mapping of pNMR to EPR tensors provides us with a more natural language for chemical analysis, as it allows the use of DFT methodologies and it rationalizes the separation of the total pNMR shift into orbital and hyperfine terms; see eq 1. We refer the reader to a recent review for a discussion of the latest theoretical understanding of pNMR theory^12^ and to examples of application of the pNMR theory to systems with higher than doublet multiplicity or low-lying excited states.^16−21^

In the case of vanishing or negligible zero-field splitting (ZFS),^22^ and when the degenerate ground state with 2*S* + 1 multiplicity is well separated from any excited states, the hyperfine contribution to the isotropic NMR shift (δ_L_^HF^) introduced in eq 1 can be related directly to the EPR quantities electronic g-tensor **g** and hyperfine coupling A-tensor **A** as follows (in Hartree atomic units):
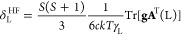
2Here, *kT* represents the thermal energy, *c* is the speed of light, and γ_L_ is the gyromagnetic ratio of nucleus L. In the following, we use the separation of δ_L_^HF^ that is governed by the physical terms of the **A**-tensor,^23^ i.e., the Fermi-contact (FC),^1,23^ paramagnetic spin–orbit (PSO),^3,23^ and spin–dipolar (SD)^23,24^ terms:
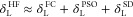
3
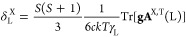
4where X in eq 4 is FC, PSO, or SD. The approximate sign in eq 3 expresses the fact that we analyze the terms on the RHS of the equation using perturbation theory (PT), where we include only the dominant terms, and that a small relativistic contribution that appears in four-component expressions is neglected.^25^ PT is most suited for chemical analysis because it allows for the use of nonrelativistic or scalar-relativistic molecular orbitals, the familiar quantity and language of chemists.

### 2.1 Hyperfine Interaction

#### 2.1.1 Fermi-Contact (FC) Mechanism

**Figure 1:**
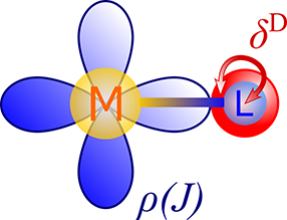
Schematic representation of the FC mechanism of the hyperfine interaction.

The Fermi-contact contribution to δ_L_^HF^ in eq 3 originates in the FC interaction between the nuclear and electron spin at the position of the nucleus (Figure 1).

Obviously, this can occur only for the imbalanced α/β ground-state electron population in the paramagnetic systems. This is analogous to perturbed electronic populations in closed-shell (diamagnetic) systems, for example, by additional nuclei with nonzero nuclear spin (FC mechanism of *J* coupling)^26^ or by spin–orbit coupling and magnetic field (SO-HALA NMR shift).^7^ One can write the FC contribution to δ_L_^HF^ through ***A***^FC^, see eq 4, as
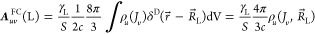
5Here, δ^D^ is a Dirac delta function,  is the position of nucleus L,  is the orientation of the magnetization (spin) of the electronic system,^12^ and ρ_*u*_ is the component of the Gordon spin density described in ref (27). Equation 5 follows from the Gordon decomposition of the current density^28,29^ and is a valid expression in four-component (4c) relativistic theory.^27^ The expression can thus easily be rewritten into a two-component (2c) or one-component (1c) theory by expressing the spin density in the respective form. Note that in this work the label 1c will encompass both nonrelativistic (NR) and scalar relativistic (SR) theories. Because of the spin–orbit coupling (SOC) interaction, the 4c ***A***^FC^ has both isotropic and anisotropic nonzero components. However, in 1c theory the ***A***^FC^ becomes isotropic, because ρ_*u*_(*J*_*v*_) = (ρ^α^ – ρ^β^)δ_*uv*_^K^, with δ_*uv*_^K^ being Kronecker delta and ρ^α,β^ being the charge densities of the α and β electrons, respectively. In such case, ***A***^FC^ becomes a pure isotropic tensor and thus contributes only to the so-called contact shift; see ref (30). Because the SR contributions usually dominate over the contributions from SOC, ***A***^FC^ is mostly isotropic and thus δ_L_^FC^ is influenced primarily by the isotropic part of the g-tensor, δ_L_^FC^ ∝ ***A***^FC,iso^**g**^iso^ (see the decomposition of δ_L_ in ref (31)).

#### 2.1.2 Paramagnetic Spin–Orbit (PSO) Mechanism

**Figure 2:**
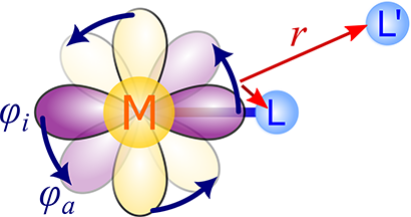
Schematic representation of the PSO mechanism of the hyperfine interaction.

The PSO mechanism originates in the interaction of the nuclear magnetic moment with the electronic orbital current density (Figure 2), which is, however, nonzero only if SOC is included in consideration.

Because the 4c expression for ***A***^PSO^ involves 4c spinors, its use in chemical analysis is nontrivial. To analyze ***A***^PSO^, one finds the perturbation theory to be a more convenient approach, utilizing the more suitable 1c MOs. The leading-order contribution to ***A***^PSO^ involves linear SOC interaction, as follows:
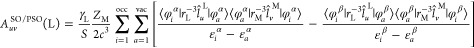
6Here, *Z*_M_ is the nuclear charge of the single heavy atom M in the system, φ_*i*_^α,β^ (ε_*i*_^α,β^) and φ_*a*_^α,β^ (ε_*a*_^α,β^) denote occupied and vacant molecular orbitals (one-electron energies), respectively, *l̂*_*u*_^X^ is a component of the angular momentum operator centered on nucleus X, and *r*_X_ is the relative distance between the electron and nucleus X with X = M, L. For a more detailed discussion of eq 6, see ref (3). Counterintuitively, the PSO mechanism contributes significantly to the long-distance limit,^32^ although one would expect that SOC would influence the electronic structure only close to the heavy atom M.

#### 2.1.3 Spin–Dipolar (SD) Mechanism

**Figure 3:**
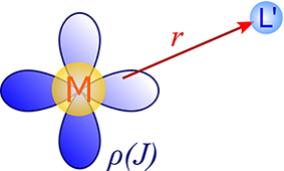
Schematic representation of the SD mechanism of the hyperfine interaction.

The SD mechanism is a consequence of the magnetic dipole–dipole interaction between the nuclear and electron spins (Figure 3).
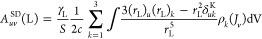
7Similar to the FC mechanism, the 4c expression in eq 7 can be derived from Gordon decomposition of the current density. In a similar way, one can reduce it to a 1c framework, where it holds ρ_*u*_(*J*_*v*_) = (ρ^α^ – ρ^β^)δ_*uv*_^K^. In such a case, ***A***^SD^ becomes purely anisotropic, and although with the inclusion of SOC ***A***^SD^ will have a nonzero isotropic part, the anisotropic part usually dominates; therefore, δ_L_^SD^ is influenced primarily by the anisotropic part of the g-tensor, δ_L_^SD^ ∝ Tr(***A***^SD,ani^**g**^ani^) (see the decomposition of δ_L_ in ref (31)). At the long-distance limit, the combined PSO and SD mechanisms lead to the well-known pseudocontact shift, which is important when investigating interactions between a paramagnetic center and a spectator nucleus L at long distances.

#### 2.1.4 Long-Distance (LD) Limit of the A-Tensor and the Hyperfine NMR Shift

Recently, it has been shown from first-principles that the long-distance limit of the A-tensor within the perturbation theory (and linear SOC effects) has the following form:^32,33^
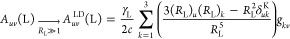
8where *R*_L_ is the distance between the paramagnetic center M and the nucleus L. Based on refs (34, 35, and 32), one can easily show that eq 8 holds even at the four-component level of theory. As expected, in the nonrelativistic limit, where the g-tensor is just the free-electron g-value g_*e*_, eq 8 gives a purely anisotropic ***A***^LD^ (L), and by inclusion of the SOC interaction, ***A***^LD^ (L) will get nonzero isotropic parts as well.

Using the LD limit of the hyperfine coupling tensor, eq 8, in the expression for the hyperfine shift, eq 2, one obtains the well-known pseudocontact shift^36,30,33,32^ that can be written as follows
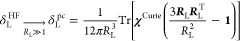
9
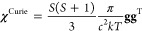
10where (***R***_L_***R***_L_^T^)_*uv*_ = (*R*_L_)_*u*_(*R*_*L*_)_*v*_ and ***χ***^Curie^ refers to the temperature-dependent part of the susceptibility tensor. Note that the LD limit in eqs 9 and 10 is also known in the literature as the point-dipole approximation.^37,36,30^ The full susceptibility tensor, ***χ*** = ***χ***^orb^ + ***χ***^Curie^, would enter eq 9 when LD is made the limit of δ_L_^tot^. However, one obtains δ_L_^pc^ from experiments as the difference between the total pNMR shift δ_L_^tot^ and its diamagnetic analogue δ_L_^orb,dia^, which approximates the orbital part of the NMR shift (δ_L_^orb^); therefore, one can write δ_L_^HF^ ≈ δ_L_^tot^ – δ_L_^orb,dia^. Note that from eq 9, one can conclude that δ_L_^pc^ arises solely due to relativistic effects, because in the nonrelativistic limit **g** = g_*e*_**1** and the tensor in round brackets is anisotropic, i.e., it has zero trace. Therefore, one must include at least linear SO effects to obtain a reasonable description of the δ_L_^pc^, and as discussed in the next section, in many cases, one must include quadratic SO effects as well.

### 2.2 Electronic g-Tensor

**Figure 4:**
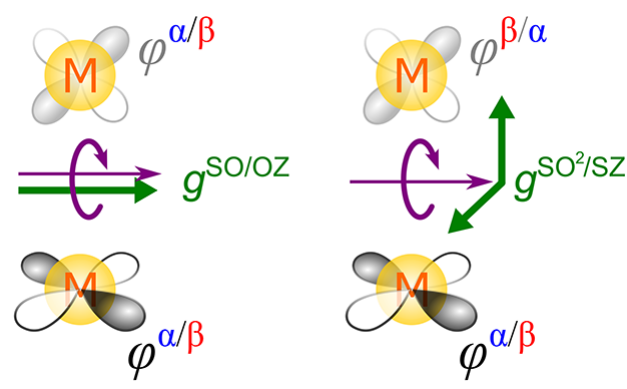
Schematic representation of the linear OZ and quadratic SZ mechanisms of the electronic g-tensor.

The final component in the EPR-based approach, as outlined in eqs 2–4, is the electronic g-tensor. The deviation of the molecular g-tensor from the g-factor of the free electron, the g-shift (Δg), originates in SOC and is governed by the orbital-Zeeman (OZ) and spin-Zeeman (SZ) mechanisms. Interestingly, the leading-order contribution is linear for the OZ interaction but quadratic for the SZ interaction. Applying perturbation theory of second and third order and accommodating some reasonable assumptions (see refs (3 and 38)) to simplify the expressions, one gets formulas useful for chemical analysis:
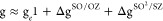
11
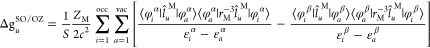
12
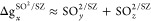
13
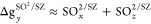
14
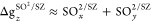
15
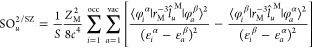
16To further simplify the discussion, we assume that the system is oriented on the principal axes of the g-tensor, i.e., only the diagonal elements of the g-tensor are nonzero. Whereas the quadratic contributions are formally *c*^–2^ smaller than the linear contributions, in heavy-element compounds, they may actually be similar to or even larger than the linear ones. Equations 12–16 reveal a useful mechanism of SOC on the g-tensor. Let us assume that there is an efficient occupied–vacant MO coupling on the metal governed by the *l̂*_*x*_^M^ operator, one can then expect sizable linear SO effects on Δg_*x*_^SO/OZ^ and quadratic SO effects on Δg_*y*_^SO^2^/SZ^ and Δg_*z*_^SO^2^/SZ^ (for a schematic representation, see Figure 4). We direct the reader to ref (38) for a detailed discussion and analysis of both linear and quadratic SO effects on the g-tensor, with general conclusions when the interplay of these effects results in a more isotropic or anisotropic g-tensor.

In Section 2 we have shown that relativistic effects (in particular, SOC) are mandatory in a correct description of pNMR shifts at short or long distances. Although relativity adds significant complexity to the problem at hand, one can apply perturbation theory, which uses only standard chemical tools, NR MOs, and angular momentum operators. One can therefore explain SO effects on pNMR shifts by extending usual chemical concepts to the relativistic domain.

## 3 Chemical Concepts

The nature of the hyperfine interaction can be used to characterize the electronic structure and geometrical arrangements of molecules and supramolecular assemblies. The electronic g-tensor and metal (M) hyperfine coupling tensor are related to the electronic structure around atom M and are pertinent to EPR spectroscopy. In contrast, weaker hyperfine interaction on distant ligand atoms (L) can be detected by NMR and gives an excellent indication of the molecular geometry and bonding.

### 3.1 “Through-Bond” Hyperfine Interaction

We start our discussion of concepts with the FC mechanism arising from the propagation of spin density from the paramagnetic center M toward the spectator atom L via chemical bonds.^39^ This includes spin delocalization based on electron conjugation (π-space) and hyperconjugation (σ-space) interactions and is further complemented by neighbor-atom or resonance spin polarization steps manifesting Hund’s rule of maximum multiplicity and Pauli’s exclusion principle.^40^

#### 3.1.1 π-Conjugation Delocalization

**Figure 5:**
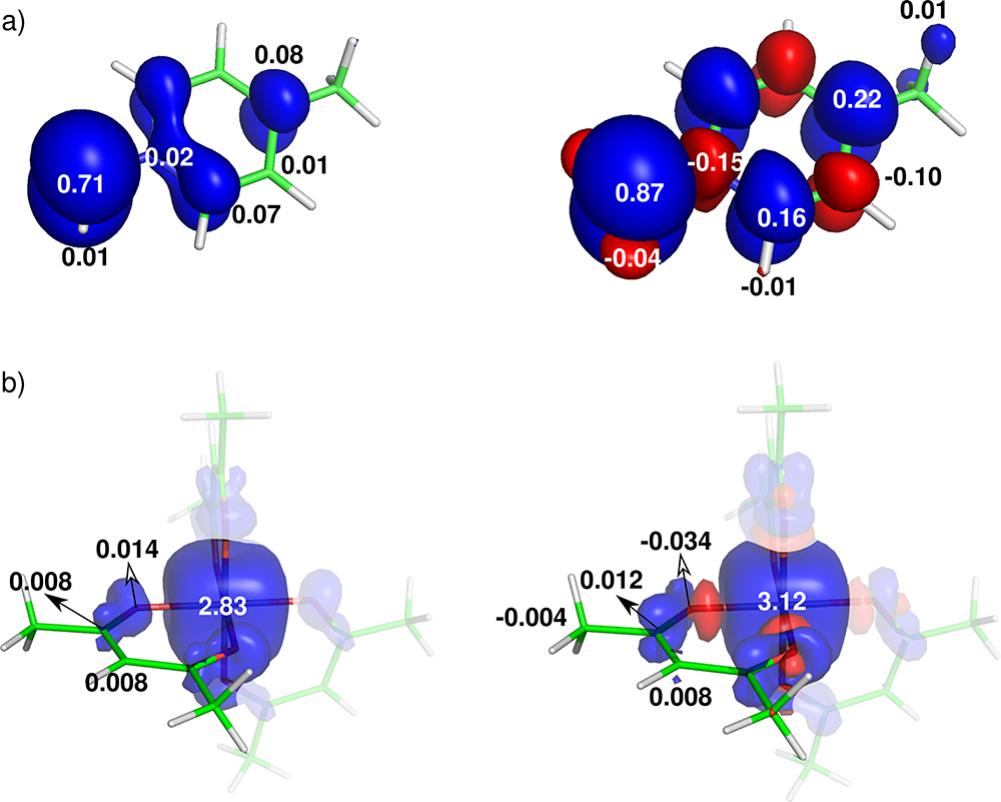
Visualization of the conjugation delocalization of α-spin density in π-space and total atomic spin populations of (a) the NMP radical and (b) Cr(acac)_3_ calculated at the spin-restricted DFT level (left) accompanied by polarization shown by the spin density and atomic spin populations calculated using the spin-unrestricted (right) approach. Adapted with permission from refs (40) and (2). Copyright 2021 and 2024 American Chemical Society.

The spin delocalization can be defined as the distribution of α-spin density over the atoms with nonzero atomic coefficients in singly occupied molecular orbital(s), SOMO(s), calculated at the spin-restricted level.^40^ This can be clearly seen in the example of the *N*-methylen-4-methyl-pyridinium radical (NMP) depicted in Figure 5a.

In the spin-restricted calculation, the α-spin density is delocalized to carbon atoms at positions *ortho* and *para* relative to the *N*-methyl group (Figure 5a). The system is polarized in spin-unrestricted calculations resulting in an increased α-spin density at positions *ortho* and *para* (Hund’s rule), and consequently, the induction of β-spin density at positions *meta* and *ipso*. In other words, the overabundance of α-spin density on atoms contributing to the SOMO in the restricted calculation (delocalization) is responsible for a further accumulation of α density on these atoms, maximizing the exchange interaction. This results in an overabundance of β density on the neighboring atoms from which the α density is pulled (polarization). The propagation of spin polarization through delocalized π-space can be explained from the perspective of perturbation theory.^41^

Analogous behavior is obtained for the Cr(acac)_3_ compound, where three unpaired electrons reside in degenerate SOMOs oriented in π-space (*d*_*xy*_, *d*_*xz*_, and *d*_*yz*_) relative to individual bonds with ligands^2^ (Figure 5b). The α-spin density in the spin-restricted calculation is delocalized from M via π-space to ligand atoms O–C with atomic coefficients contributing to the SOMO. In the spin-polarization step enabled in the spin-unrestricted calculation, the metal center M pulls further α density predominantly in σ-space (see Neighbor-Atom and Resonance Polarization below) because of the symmetry of the SOMOs, thus leaving an overabundance of β density on the M–O bond.

#### 3.1.2 σ-Hyperconjugation Delocalization

**Figure 6:**
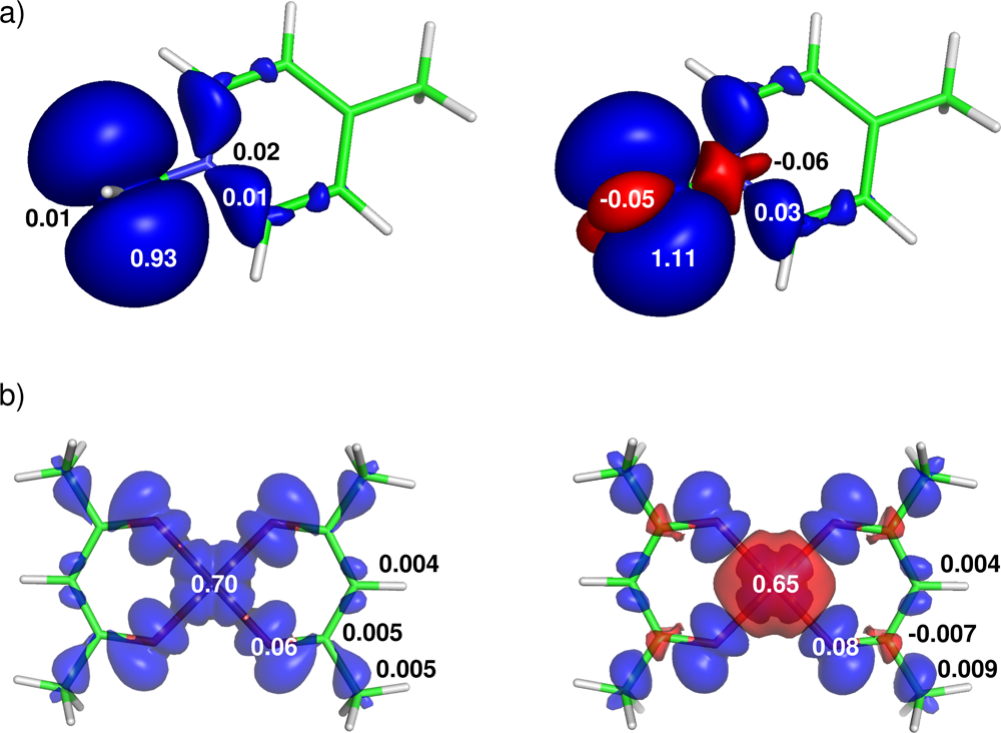
Visualization of the hyperconjugation delocalization of the α-spin density in σ-space and the total atomic spin populations of (a) the transition state of NMP and (b) Cu(acac)_2_ calculated at the spin-restricted DFT level (left), with the polarization shown at the spin-unrestricted approach (right). Note the β-spin polarization of the outer shell of the Cu atom.^42,43^ Adapted with permission from refs (40) and (2). Copyright 2021 and 2024 American Chemical Society.

The situation is different for systems with different symmetries of the SOMO relative to the pyridine π-space and the absence of its conjugation. This applies to the NMP with a CH_2_ group rotated by 90° in the transition state^40^ shown in Figure 6a.

Here, the α-spin density in the restricted calculation is delocalized to positions *ortho* (and *meta*) of the aromatic ring via σ-hyperconjugation. As a result of the spin polarization in the unrestricted calculations, these atoms accumulate additional α density and leave β density at the neighboring position *ipso*.

An analogous situation is observed for the square-planar Cu(acac)_2_ compound, Figure 6b, where the α-spin density in the SOMO (*d*_*x*^2^–*y*^2^_) oriented in the M–L bonds is delocalized to the carbons of the CH_3_ groups via σ-hyperconjugation^2^ (see also σ delocalization in the high-spin Fe^2+^ complex^20^). The spin polarization in the unrestricted calculation induces β density at the position of the carbonyl carbon.

#### 3.1.3 Neighbor-Atom and Resonance Polarization

**Figure 7:**
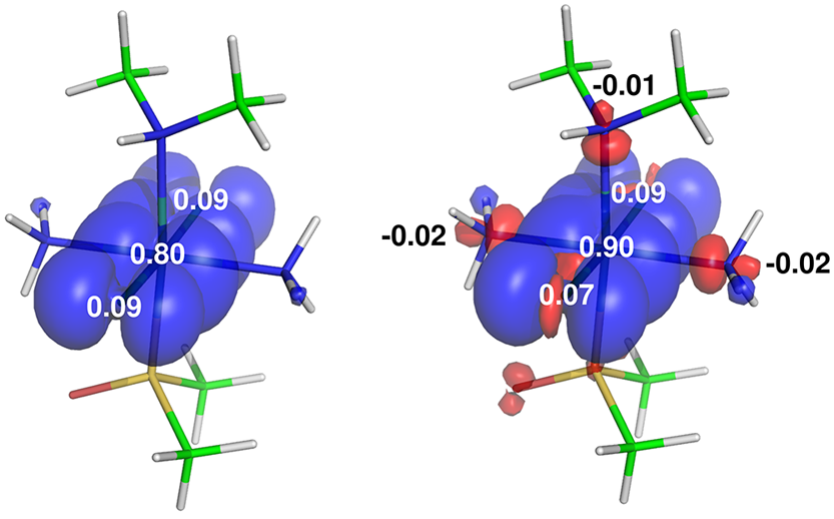
Visualization of the spin density and atomic spin populations on the M and neighboring ligand atoms in the model [RuCl_2_(NH_3_)_2_(NHMe_2_)(DMSO)]^+^ calculated at the restricted (left) and unrestricted (right) DFT levels. Adapted with permission from ref (40). Copyright 2024 American Chemical Society.

In the absence of π-conjugation or σ-hyperconjugation delocalization discussed above, the only mechanism of spin transmission is polarization.^40^ The presence of α density at M results in pulling additional α density to M in the unrestricted calculation (Figure 7).^43^ It leaves β density at the neighboring atoms as a manifestation of Pauli’s principle.^44^ This neighbor-atom polarization can propagate further in a consecutive manner, although the σ-polarization pathway is inefficient because of the “hard” nature of σ bonds compared to the “soft” nature of the π system discussed above.^45^ Alternative mechanisms involve σ or π resonance polarization as described in ref (40).

#### 3.1.4 Solvent Effects

**Figure 8:**
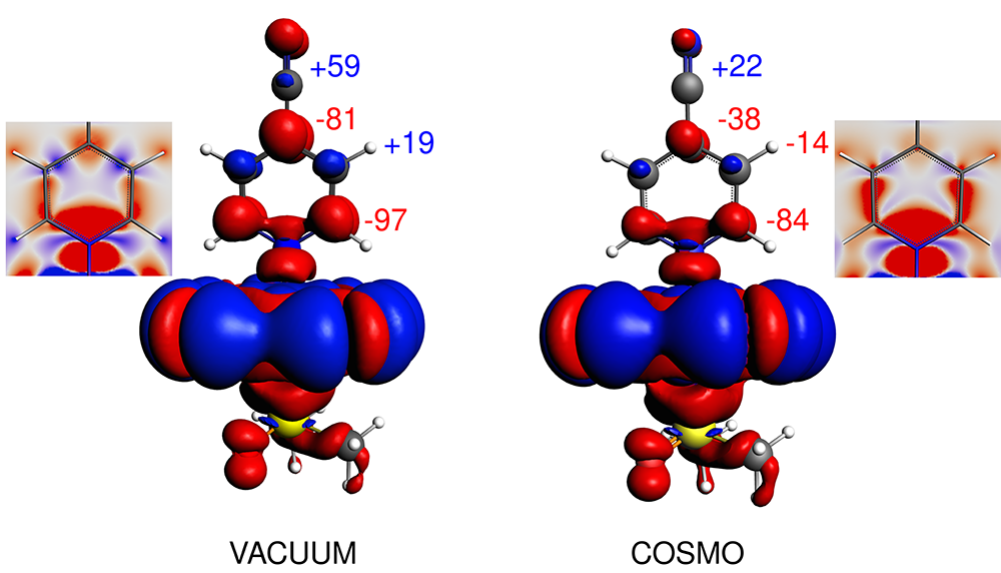
Visualization of the spin density and hyperfine shifts for carbon atoms of the pyridine in Na^+^[Ru^III^Cl_4_(4-CN-pyridine)(DMSO)]^−^ calculated *in vacuo* (left) and using an implicit solvent model (right). The in-plane spin polarization of the pyridine is represented as a detailed slice of the spin density at the lower threshold (0.00005 au). Adapted with permission from ref (1). Copyright 2016 American Chemical Society.

We demonstrated that the efficiency of σ vs π resonance polarization is greatly influenced by the solvent.^1,23^ This can even result in switching the sign of the FC mechanism,^1^ as shown in the example of the *meta* carbon (+19 ppm vs −14 ppm; visualization of the π and σ resonance polarizations in Figure 8) of the pyridine in the Ru(III) complex. Therefore, the calculation of solution-state data should preferably be done using at least an implicit solvent model.

#### 3.1.5 Crystal Packing Effects

**Figure 9:**
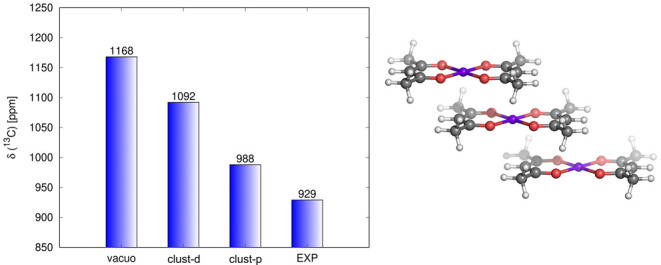
^13^C NMR shift of the methyl group in Cu(acac)_2_ calculated *in vacuo* (vacuo), in a cluster with two diamagnetic neighbors (clust-d), in a cluster with two paramagnetic neighbors (clust-p), and the experimental value (exp). Adapted with permission from ref (2). Copyright 2021 American Chemical Society.

Supramolecular interactions have large effects on the hyperfine shifts in the solid state, where neighboring molecules not only influence the electronic structure of the investigated system but also can be sources of additional hyperfine fields (spin centers that create additional through-space hyperfine interactions). This can be exemplified by the ^13^C NMR of the methyl group calculated *in vacuo*, in the crystal environment approximated by the two diamagnetic neighbors (cluster-d), and in the environment of the two paramagnetic neighbors (cluster-p)^2^ (Figure 9).

The ^13^C NMR shift of +1168 ppm calculated *in vacuo* is reduced by approximately 80 ppm in the presence of two diamagnetic neighbors. Further, recovering the electronic doublet character of each of the two neighboring molecules results in an additional shielding of more than 100 ppm, thus reaching the value of 988 ppm (the experimental resonance at 929 ppm).^2^

### 3.2 “Through-Space” Hyperfine Interaction

In the absence of electron sharing or charge transfer between the atoms or fragments discussed in Section 3.1, the hyperfine interaction propagates exclusively “through-space”. This arises from the PSO and SD mechanisms, and at long distances corresponds to the pseudocontact shift (see Section 2.1.4).^46^ However, the through-space interaction can dominate even at short distances in compounds with ionic or only weakly covalent bonds lacking notable electron sharing (inefficient FC mechanism; e.g., *f*-block lanthanide complexes).^47^ However, in *d*-block TM complexes, these short-range shifts are dominated by the FC term. Therefore, only through-space effects at longer distances are discussed further for the TM compounds.

#### 3.2.1 Through-Space Shift via Full Expression in Equation 2

**Figure 10:**
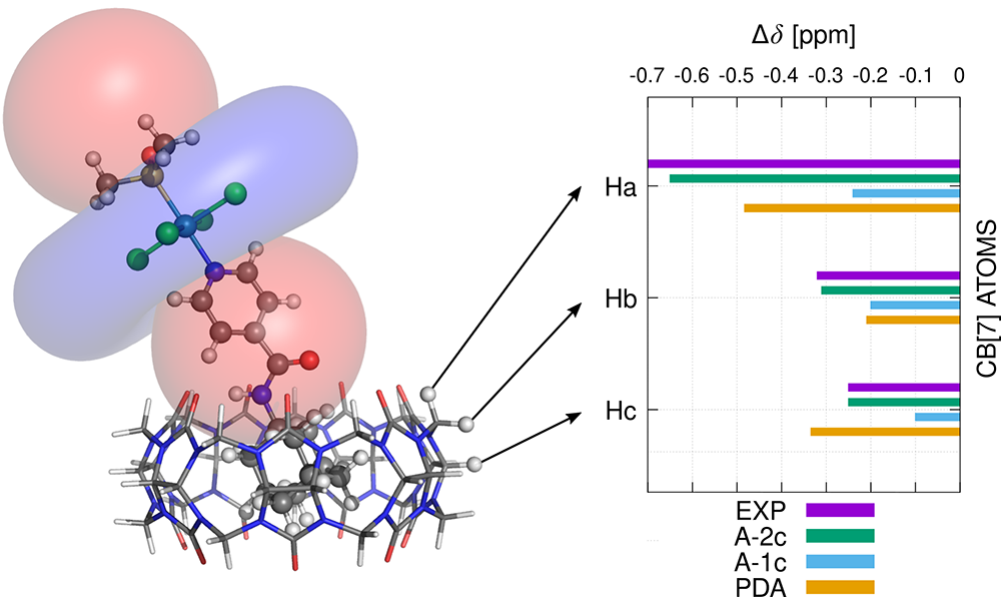
Hyperfine ^1^H NMR shifts of a CB7 host obtained experimentally (EXP) and calculated by DFT: 2c **g** and **A** values (A-2c), 2c **g** and 1c **A** (A-1c), and using PDA. Data from ref (24).

The contributions of the PSO and SD to the hyperfine shift can be calculated at the 2c or 4c level of theory using eq 2 involving both g- and A-tensors. However, the size of the system may prohibit the usage of 4c and even 2c computational methods for full systems. In the past, various methods have been tested to find approximate and computationally more feasible approaches. For example, only the surroundings of M have been used to calculate the g-tensor at the 2c level, and the distant L hyperfine coupling has been obtained at the 1c level for the full system.^24^ Although such a calculation yields artificially small values due to the missing contributions from the PSO, the trends in δ_L_^HF^ remain intact in systems with small g-tensor anisotropy (Section 2),^24,48^ as shown in Figure 10.

#### 3.2.2 Through-Space Shift at Long Distances

At long distances, through-space hyperfine shielding can be calculated from the less expensive magnetic susceptibility tensor obtained via the electronic g-tensor. Note that this approach has been termed the long-distance (LD) limit or point-dipole approximation (PDA).^32,49,30^ In practice, it applies to situations where the Fermi-contact contribution is vanishingly small and the spatial distribution of spin density around M relative to L can be neglected (for applications, see Section 4.2).

## 4 Applications

### 4.1 Through-Bond: Electronic Structure and the FC Term

#### 4.1.1 Bond between M and L

The character of bonding along the transmission pathway from M is crucial for the spin delocalization and polarization toward the ligand L. The degree of covalency (electron sharing) can be characterized using regular chemical tools, for example, by the delocalization index (QTAIM)^50^ or NOCV channels.^1,23^

**Figure 11:**
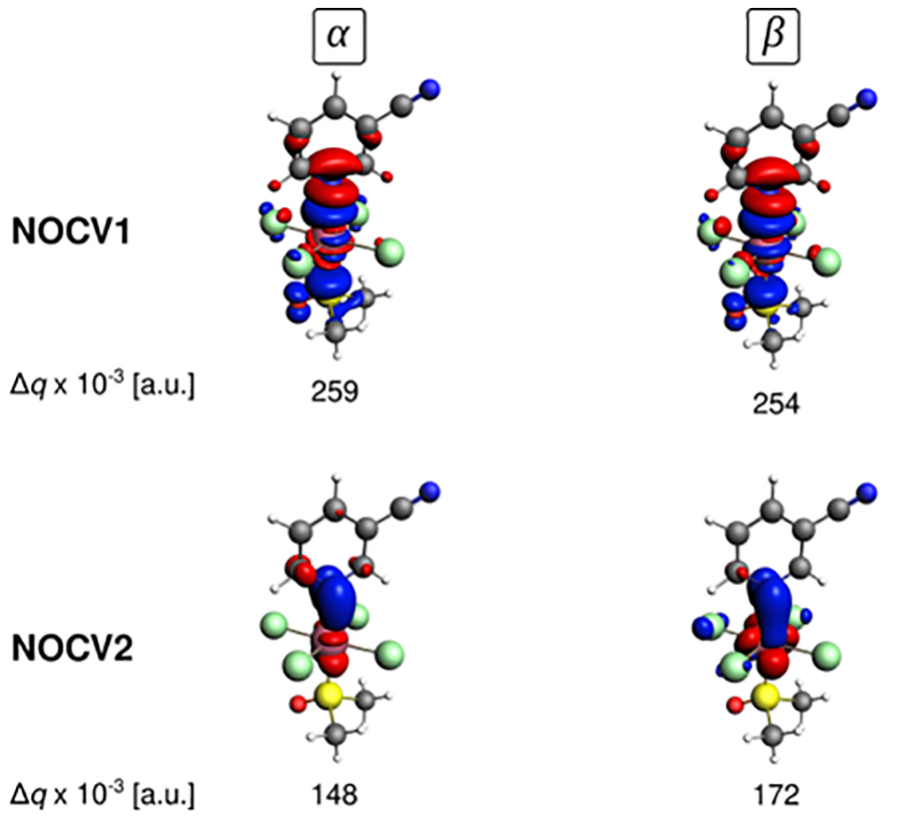
Visualization of the two most important NOCV channels (α and β parts) for the formation of the Ru–N bond. The value 0.5 × 10^–3^ au was used to plot the isosurface of the electron deformation density. Adapted with permission from ref (23). Copyright 2018 American Chemical Society.

In prototypical Ru(III) compounds^51^ with pyridine ligands, concepts of σ-donation and π-backdonation have been used to describe the bond between the ruthenium and nitrogen atoms (Figure 11).^23^ Note that the spin delocalization is quenched in this arrangement because of the unfavorable symmetry of the SOMO.^40^ The first σ-channel represents a classical donation of the lone pair of electrons of the nitrogen atom to M. This is slightly more efficient in the α-space (Hund’s rule), thus leaving an overabundance of β density in the σ-space of pyridine. The second π-backdonation channel is more efficient in β-space, thus leaving α density at M and transmitting β density toward the *ortho* and *para* positions of the π system.

#### 4.1.2 Effect of a *trans* Ligand Attached to M

**Figure 12:**
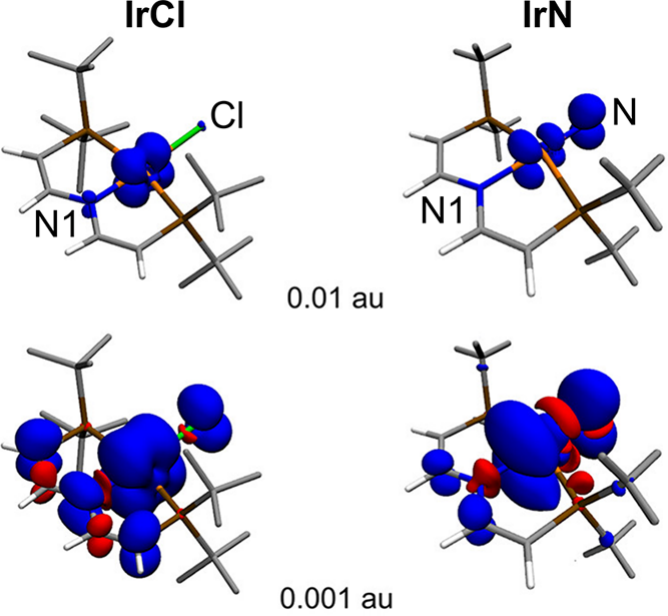
Visualization of the spin density in compounds **IrCl** (left) and **IrN** (right), highlighting the switch in the transmission mechanism induced by the *trans* ligand and the oxidation state of the Ir atom. Adapted with permission from ref (3). Copyright 2019 American Chemical Society.

The character of the ligands can influence the distribution of spin density around the magnetic M and the transmission of spin density toward the spectator atom(s). This can be classified as a *cis* or equatorial-to-axial ligand effect (weak) involving mostly neighbor-atom polarization and a hyperfine *trans* effect (strong)^40^ with two ligands competing for one *d* orbital (e.g., *d*_*z*^2^_) of the M.^52^ This leads to changes in the M–L bond distance and covalency upon the change of the *trans* ligand and simultaneously modulates the mechanism of the spin transmission and the ligand hyperfine coupling, as shown by the example of the iridium compounds in Figure 12. Because of the relatively weak bond between the *trans* chloride and the iridium in compound **IrCl**, the Ir–N1 bond is strong, with significant delocalization of the spin density into the π-space of N1. This is propagated further with altered signs, as described for conjugated systems in Section 3.1. In contrast, because of the strong triple bond between the *trans* nitrogen and iridium in compound **IrN**, the Ir–N1 bond is relatively weak, hindering direct spin delocalization. Therefore, the spectator atom N1 is spin-polarized in the π-space and the resulting spin-density pattern around the N1–C2–C3 fragment in **IrN** is the inverse of that in **IrCl**.

#### 4.1.3 Effect of Substituents on the Aromatic Ligand

**Figure 13:**
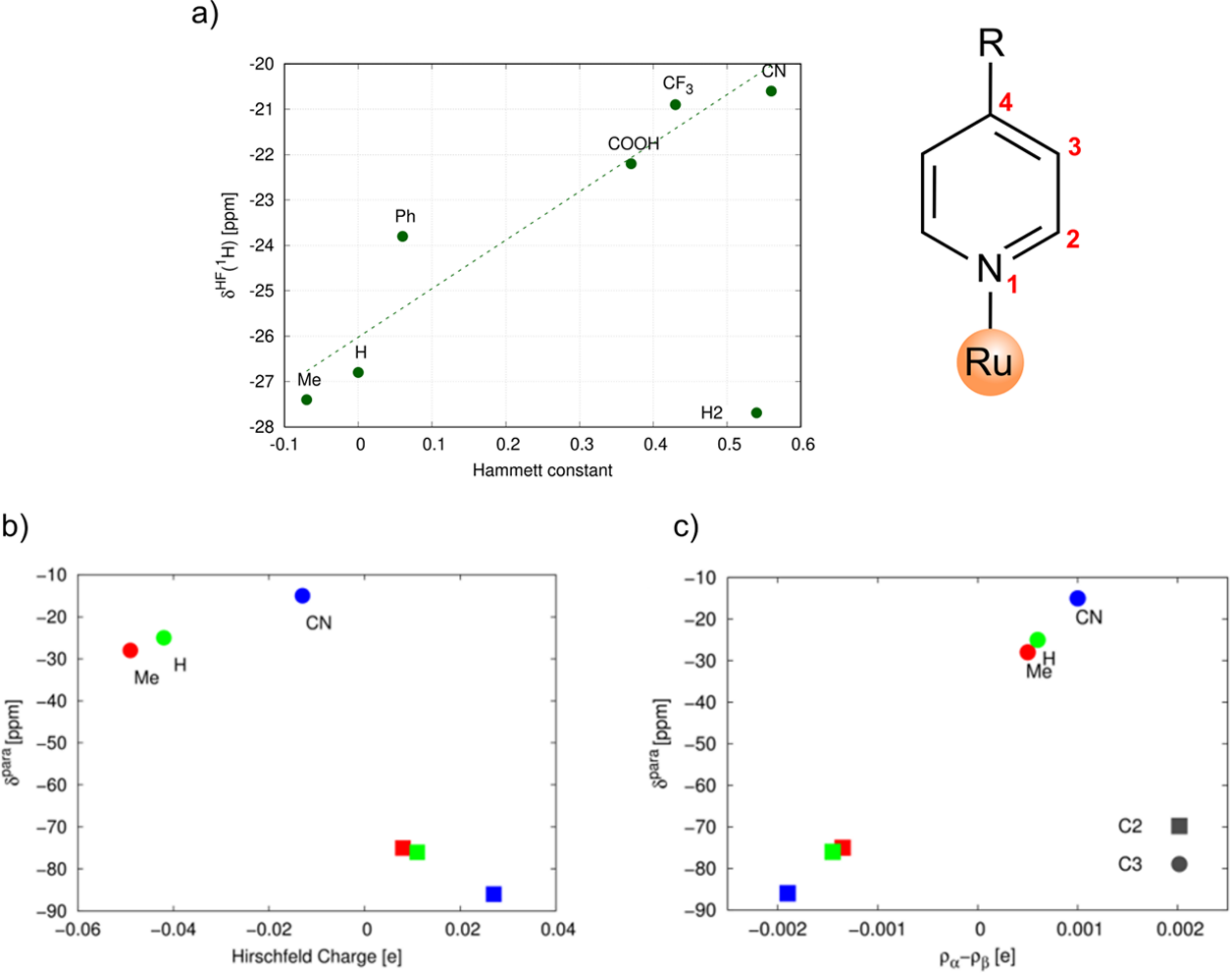
Correlation of the (a) Hammett constant, (b) Hirschfeld charge, and (c) atomic spin population with the hyperfine ^1^H or ^13^C NMR shift. Adapted with permission from refs (52) and (1). Copyright 2016 and 2023 American Chemical Society.

The character (electronic properties) of the substituents influences the distribution of electron density on the ligand and by that also the spin transmission from M toward the ligand atoms. The effect of the substituent on the electron (and spin) density is shown by correlating the δ_L_^HF^ with the Hammett constant,^52^ Hirshfeld charge,^1^ and atomic spin population^1^ in Figure 13. Depending on the position of the electron donor (acceptor), the concentration (depletion) of the atomic electron density can enhance or diminish the spin polarization and the FC contribution to δ_L_^HF^.

### 4.2 Through-Space: Host–Guest Assemblies and the SD and PSO Terms

#### 4.2.1 NMR Regime of Slow Chemical Exchange

**Figure 14:**
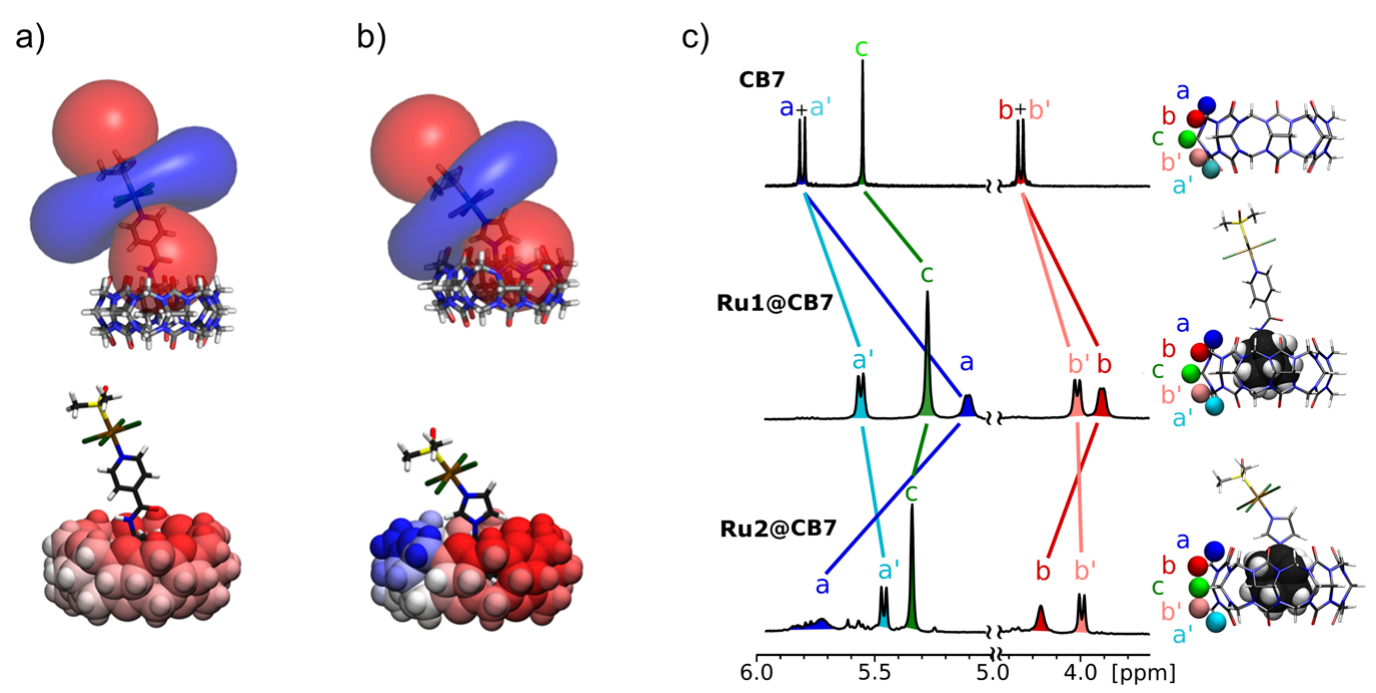
Visualization of the hyperfine deshielding (blue) and shielding (red) regions around the molecules of compounds (a) **Ru1** and (b) **Ru2** in their HG assemblies with CB7.^24^ (c) NMR spectra of free CB7, **Ru1@CB7**, and **Ru2@CB7**. Adapted with permission from ref (24). Copyright 2018 American Chemical Society.

Strong host–guest (HG) complexes give NMR resonance lines resolved from those of the free host and guest components. Therefore, the interpretation of their pNMR spectra is straightforward and can be used to determine the relative HG distance and orientation. It has been shown^24^ that the ruthenium guest **Ru1** with the pyridine linker to an adamantyl anchor has a distance from the oxygen portal of cucurbit[7]uril (CB7) of 763 pm, whereas this distance is much shorter (469 pm) for the guest **Ru2** with an imidazole linker. This would imply stronger through-space effects and a larger δ_L_^HF^ in **Ru2**. Indeed, this has been observed for the theoretically calculated values visualized in Figure 14. However, because of the larger tilting (26° vs 37°) of the guest inside the host, both shielding and deshielding hyperfine effects on the equivalent atoms of CB7 are observed for **Ru2**. As a result of the HG dynamics in solution (rotational tumbling), the shielding–deshielding effects are almost averaged out, and the experimentally observed complexation δ_L_^HF^ for hydrogen Ha in **Ru2** is negligible (Figure 14). In other words, whereas the portal atoms Ha and Ha′ in **Ru1** are shielded upon complexation as expected, Ha in **Ru2** is shifted only marginally because the shielding and deshielding effects are averaged.

#### 4.2.2 NMR Regime of Fast Chemical Exchange

**Figure 15:**
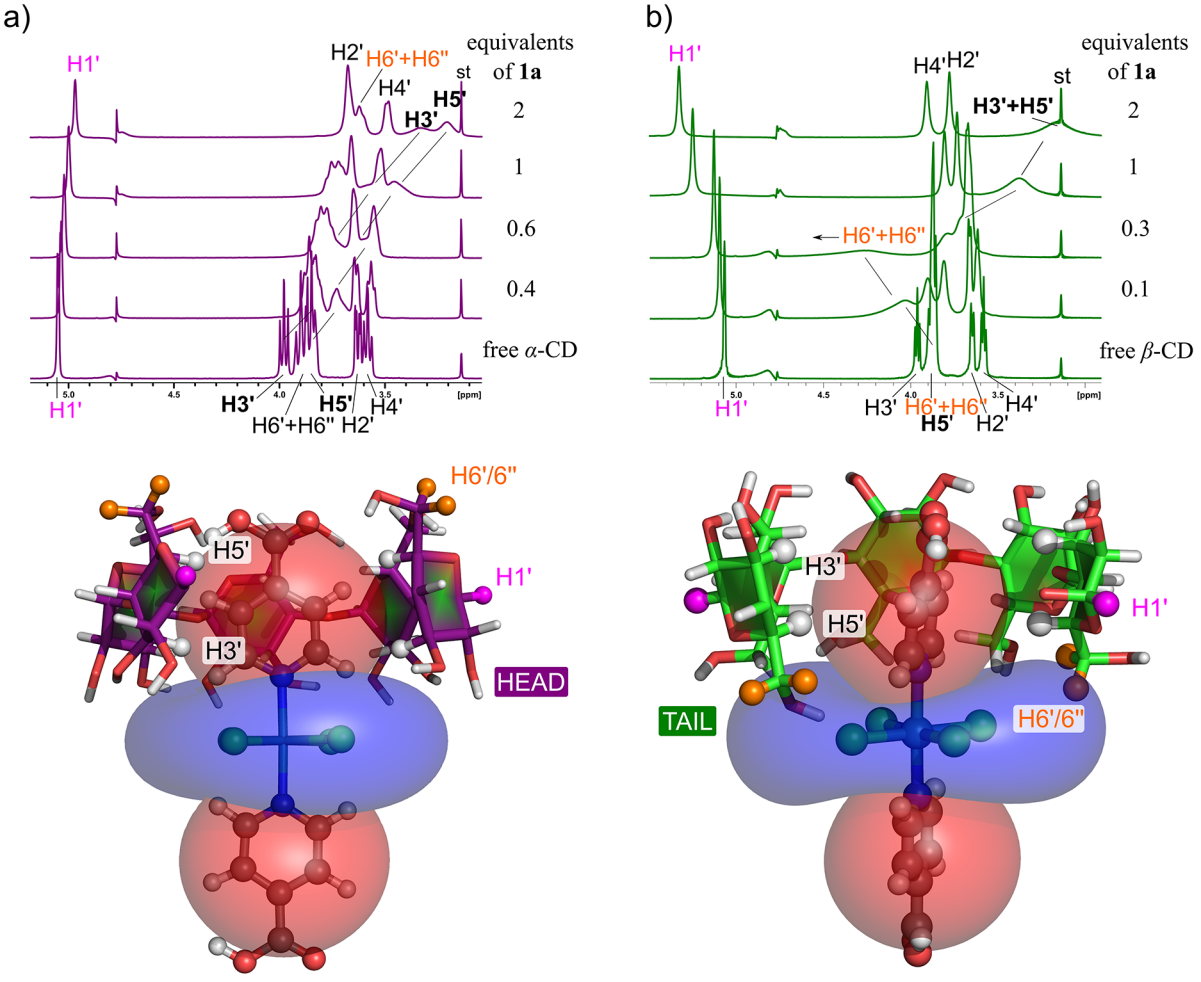
Visualization of the hyperfine deshielding (blue) and shielding (red) regions around the molecule of compound **Ru3** in its host–guest assembly with (a) α-CD, head and (b) β-CD, tail.^4^ Reproduced from ref (4). Available under a CC BY 4.0 license. Copyright 2023 Jan Novotny and Radek Marek.

In the fast-exchange regime, both the free and bound forms of the guest and host contribute to the (population-weighted) averaged NMR signals. Therefore, the averaged signals are affected by various factors, such as the HG affinity (binding constant and free–bound ratio), the geometry of the HG assembly, and the magnetic anisotropy of the paramagnetic center. pNMR spectroscopy has been used to characterize the relative position of the cyclodextrin (CD) host in the hyperfine field of the ruthenium guest.^4^ The NMR spectra for CD at various HG ratios with compound **Ru3** and the calculated hyperfine shielding maps are shown in Figure 15. This approach allowed the determination of head versus tail HG binding.

## 5 Summary and Outlook

The NMR spectroscopy of paramagnetic systems has developed as an extremely powerful technique in investigating hyperfine interactions. In this Account, we build on the separation of the hyperfine shift into “through-bond” and “through-space” contributions that are associated with essential physical hyperfine mechanisms. We aim to discuss these mechanisms, Fermi-contact (FC), paramagnetic spin–orbit (PSO), and spin–dipolar (SD), for a general chemical readership employing standard chemical concepts involving molecular orbitals. We provide several models to demonstrate the principles of spin delocalization and polarization in σ- and π-space to rationalize the distribution patterns of the spin density in ligands. We discuss the effects of the solvent and environment on the Fermi-contact mechanism of hyperfine shifts. Further, we demonstrate the applicability of long-range hyperfine interactions (PSO and SD) to determining the geometry of supramolecular host–guest complexes.

Even after decades of progress in interpreting NMR spectra for both diamagnetic and paramagnetic substances, the interpretation of pNMR spectra still lags behind that of diamagnetic NMR. Obviously, this is due to the inherent physical complexity underlying the pNMR spectra. In contrast to the NMR shifts of diamagnetic compounds, pNMR shifts are influenced by multiple nonadditive interactions characterized by nonlocal character and strong dependence on relativistic effects. Our Account is a step toward a deeper understanding of the chemical concepts underlying hyperfine interaction and its manifestation in pNMR spectra.
